# A preliminary study on the characteristics of Th1/Th2 immune response in cerebrospinal fluid of AIDS patients with cryptococcal meningitis

**DOI:** 10.1186/s12879-021-06138-z

**Published:** 2021-05-29

**Authors:** Aixin Li, Wenjiao Zhu, Jiming Yin, Xiaojie Huang, Lijun Sun, Wei Hua, Wen Wang, Tong Zhang, Lili Dai, Hao Wu

**Affiliations:** 1 Center for Infectious Diseases, Beijing Youan Hospital, Capital Medical University, Beijing, 100069 China; 2 Department of Dermatology, The Affiliated Hospital of Qingdao University, Qingdao, 266003 China; 3 Beijing Institute of Hepatology, Beijing Youan Hospital, Capital Medical University, Beijing, 100069 China

**Keywords:** Acquired immunodeficiency syndrome, Cryptococcus, Cryptococcal meningitis, Cerebrospinal fluid, Cytokines

## Abstract

**Background:**

Cryptococcal Meningitis (CM) is a common opportunistic infection in the late stage of acquired immunodeficiency syndrome (AIDS). Despite the wide use of effective antiretroviral and antifungal therapy in AIDS patients, CM is still a major morbidity and mortality cause. Understanding the immune response in cryptococcal infection may help to improve the treatment strategies.

**Methods:**

We established a prospective cohort of twelve AIDS patients with CM (HIV + CM+) admitted to the hospital from 2019 to 2020. All patients were examined at the baseline, 2 weeks, and 4 weeks thereafter. The level of 19 cytokines in cerebrospinal fluid (CSF) were recorded to analyze the characteristics and dynamic changes of Th1/Th2 immune response. Meanwhile, six AIDS patients without CM (HIV + CM-) and seventeen healthy subjects (HIV-CM-) were included as control groups for CSF assessment.

**Results:**

The HIV+ CM+ group had higher CSF IFN-γ, TNF-α, IL-6, IL-7, IL-8, IL-10, IL-12 (P40), IL-15, IL-18, CCL2 levels but lower IL-4 when compared with the HIV-CM- group at baseline. And they also had a higher level of IL-12 (P40) and IL-17A compared with HIV + CM- patients. Except one patient dropped out of the study, eleven HIV + CM+ patients received induction antifungal therapy and regular CSF testing, and the mortality rate was 9.1% (1/11) and 18.2% (2/11) respectively at week 2 and week 4. Compared with baseline CSF cytokines, IL-2, IL-13, IL-17A, and VEGF-A decreased in week 2, and the VEGF-A levels further decreased in week 4. But there was no difference in the levels of all cytokines between survivors and the dead.

**Conclusion:**

No evidence of Th1/Th2 imbalance was found in AIDS patients with CM. However, the CSF cytokine network may provide new clues for the treatment of AIDS patients with CM.

**Trial registration:**

This trial was prospectively registered in 2019.7.16. The registered number is ChiCTR1900024565.

**Supplementary Information:**

The online version contains supplementary material available at 10.1186/s12879-021-06138-z.

## Background

Cryptococcal meningitis (CM) is a common opportunistic infection in the late stage of acquired immunodeficiency syndrome (AIDS), which causes the second most AIDS-related death after tuberculosis [1, 2]. A recent study of the global burden of cryptococcal disease estimated that 278,000 individuals have a positive cryptococcal antigen test which indicates the infection, and 223,100 patients develop CM [1]. Although CM has been effectively controlled with the widespread application of antiretroviral therapy (ART) and antifungal drugs, the mortality rate of CM in AIDS patients remains high ranging from 30 to 50%, especially for patients in low-resource settings [3, 4].

The onset of CM is often insidious with atypical clinical manifestations in AIDS patients. The treatment success rate is low [5], and it is prone to relapse. It is yet to determine what factors affect the treatment response and clinical outcome. With few new antifungal drugs being developed [6], cryptococcal immunotherapy, aiming at regulating the immune response and improving the prognosis [7, 8], has gained attention. Increasing evidence showed that the host’s immune response to cryptococcosis plays an essential role in the manifestation and prognosis [9–11]. However, the current understanding of cryptococcal immune response is mainly derived from in vitro and animal experiments. Animal model studies show that the immune response of type 1 T helper cells (Th1) is protective, while that of type 2 T helper cells (Th2) is mainly non-protective, even harmful [12–14]. Supportive data in human hosts are limited and seem to support more complex effector T cell responses that differ from animal models [15, 16]. A study indicated that high serum pre-ART levels of IL-4 and IL-17 were predictive of future immune reconstitution inflammatory syndrome (IRIS) in AIDS patients with CM [17]. In contrast, Zheng’s research showed that decreased IL-4 and IL-17 levels were associated with high intracranial pressure (ICP) during IRIS [18]. These paradoxes indicated that serum cytokines themselves required further investigation. In addition, studies also showed that due to the cytokines levels in CSF not always corresponding to the level of serum [19, 20], it implied local factors might play an important role in the central nervous system disease.

In this study, we aim to prospectively evaluate the concentration changes of CSF cytokines in AIDS patients with CM and examine the relationship of CSF cytokines with clinical characteristics and prognosis.

## Materials and methods

### Study population and diagnosis

**Fig. 1:**
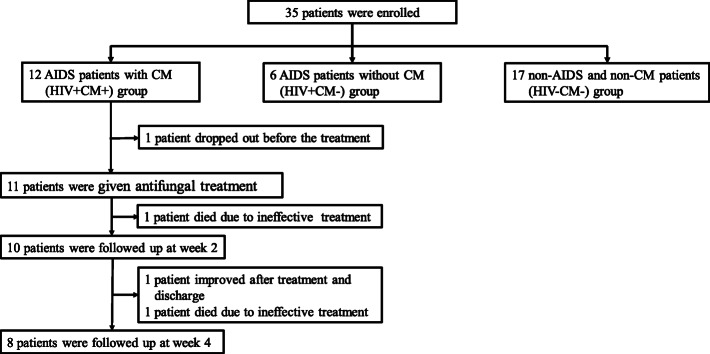
The flow chart of subjects recruiting

This prospective study enrolled 12 AIDS patients with CM (HIV + CM+) at Beijing Youan Hospital from 2019 to 2020 (Fig. 1). The inclusion criteria were 1) 18–65 years old patients of all sex, 2) diagnosed with HIV and CM [21], and 3) ART naive at enrollment. All patients were confirmed with anti-HIV-1 antibody screening by enzyme-linked immunosorbent assay (ELISA) and Western blotting test (WB). CM’s diagnostic criteria include positive CSF India ink stain, CrAg test, and *Cryptococcus neoformans* culture. CSF was used as the primary index because in the preliminary experiment, no association was found between cytokines in plasma and CSF (Fig. S1).

Two control groups were established. The first group consisted of 6 AIDS patients without CM (HIV + CM-) matched by age and CD4+ T cells count. The non-CM infections are including simple cryptococcal antigen-positive, tuberculosis, or syphilis. Diagnostic lumbar puncture was required for these patients with a CSF aliquot collection, which confirmed the absence of CM. A second control group consisted of 17 non-AIDS and non-CM patients (HIV-CM-), who was admitted due to syphilis or non-infectious neurological disorders such as migraine or tension-type headache also with the absence of CM by CSF aliquot test.

### Treatment and clinical outcomes

All HIV + CM+ patients were given antifungal treatment after admission. Induction therapy contains minimal 2 weeks amphotericin B deoxycholate (0.7–1.0 mg/kg/day) and fluconazole (800 mg/day), until getting clinical symptoms alleviated and CSF sterile. It was followed by a consolidation therapy using minimal 8 weeks of fluconazole (400 mg/day), then maintained by fluconazole 200 mg/day. Antiretroviral treatment was initiated at 4 to 6 weeks post commencement of antifungal therapy. HIV + CM- patients who were simple cryptococcal antigen-positive were given fluconazole 400 mg/day antifungal therapy. The endpoint was the 2-week and 4-week mortality.

### Laboratory tests

All patients underwent baseline blood CD4+ T cell counts, and HIV+ patients underwent Plasma HIV-1 RNA concentrations testing at enrollment. Absolute blood CD4+ T cell counts (cells/μl) were measured using a FACS Calibur flow cytometry (BD, Franklin Lakes, New Jersey, USA). Plasma HIV-1 RNA concentrations (copies/ml) were quantified using the COBAS AMPLCORTM HIV-1 Monitor v1.5 or COBAS Ampliprep/COBAS TaqMan 48 Analyzer (Roche Diagnostic, Branchburg, New Jersey, USA), with a detection limit of 40 copies/ml of plasma.

CSF of all patients was obtained at the admission as a baseline. The operation of lumbar puncture was performed by an experienced clinician, and the CSF was collected, avoiding the first tube. In HIV + CM+ group, CSF was collected every 2 weeks during treatment. All CSF samples were frozen and stored at − 80 °C for subsequent analysis. Cytokine concentrations were measured using a commercial, human-specific, antibody-coated microsphere-based multiplex cytokine immunoassay, which was able to quantify 19 cytokines contemporaneously by using 25ul CSF (Human Cytokine/Chemokine/Growth Factor Panel A Magnetic Bead Panel, HCYTA-60 K-PX48, EMD Millipore Corporation, Billerica, MA 01821 USA).

The cytokines/chemokines/growth factors included in this kit were as follows:

Interferon-γ (IFN-γ), Interleukin-1β (IL-1β), Interleukin-2 (IL-2), Interleukin-4 (IL-4), Interleukin-5 (IL-5), Interleukin-6 (IL-6), Interleukin-7 (IL-7), Interleukin-8 (IL-8), Interleukin-10 (IL-10), Interleukin-12 p40 (IL-12 (p40)), Interleukin-13 (IL-13), Interleukin-15 (IL-15), Interleukin-17A (IL-17A), Interleukin-18 (IL-18), Interferon- γ produced protein-10 (IP-10, in the systematic nomenclature, CXCL10), Monocyte Chemoattractant Protein-1 (MCP-1, in the systematic nomenclature, CCL2), Macrophage Inflammatory Protein-1α (MIP-1α, in the systematic nomenclature, CCL3), Tumor Necrosis Factor-α (TNF-α), and Vascular Endothelial Growth Factor A (VEGF-A). The standards and quality controls for all samples were assayed following the instructions of the manufacture. Two hours of incubation at room temperature and a magnetic plate washer were utilized. The plates were read with a multiplex plate reader and companion software. All cytokine concentrations were reported in pg/ml. If it were below the lower limits of detection, the concentration level would be reported as the midpoint between zero and the lowest concentration of each cytokine measured.

### Statistical analysis

Continuous variables with abnormal distribution were analyzed with the Kruskal-Wallis H-test, described as the median and interquartile range (IQR). Categorical variables were expressed as the number of cases (percentage) and compared by Fisher’s exact test. Generalized estimating equations were used to analyze the association between CSF cytokines and treatment time or clinical outcome. The correlation among CSF cytokine concentrations for HIV + CM+ group, HIV + CM- group and HIV-CM- group were determined using Spearman correlation coefficients. Correlation matrices were displayed as schematic correlograms. Statistical analyses were performed using SPSS 25.0 statistical software (SPSS Inc., IL, USA), GraphPad 8 (GraphPad Software, California, USA), and open-source procedure R 3.6.1 (https://www.r-project.org). A *P* value < 0.05 (two-tailed) was considered statistically significant.

## Results

### Demographics of participants

Among 12 HIV + CM+ patients, 10 (83%) were male and 2 (17%) were female with the mean age of 38.5 (34.5, 46.5) years old at the baseline. Clinical features of fever 7 (58%), headache 8 (67%), nausea 4 (33%), stiff neck 5 (42%), altered mental status 4 (33%), pathological signs 1 (8%), and increased ICP 7 (58%) were observed upon admission. The median time of HIV infection detection was 30 days (4.5, 165.0), while the onset of symptoms to hospitalization was 12.5 days (0.5, 30.0). The lymphocyte count and serum albumin level in the HIV-CM- group were significantly higher compared to the HIV + CM+ group (*P* < 0.001) and the HIV + CM- group (*P* < 0.001) (Table 1). In HIV + CM+ group, the CSF white blood cell (WBC) count and lymphocyte count were higher than those in the HIV + CM- group (*P* = 0.008 and *P* = 0.003) and HIV-CM- group (*P* = 0.011 and *P* = 0.016). While the CSF chloride in the HIV + CM+ group was lower compared to the HIV-CM- group (*P* = 0.008) (Table 1).

**Table 1:** Baseline characteristics of the three groups

Parameters	HIV + CM+ (***n*** = 12)	HIV + CM- (***n*** = 6)	HIV-CM- (***n*** = 17)	***P*** value
*Demographics*				
Age (years)	38.5 (34.5,46.5)	41.5 (34.0,54.3)	38.0 (28.5,49.5)	0.689
female, n (%)	2 (17)	1 (17)	12 (71)	0.005
*Laboratory exam*				
WBC count(×10^9/L)	5.4 (3.4,9.0)	4.8 (2.4,6.2)	5.6 (4.5,6.5)	0.543
Lymphocyte count (×10^9/L)	0.5 (0.3,0.7)	0.8 (0.6,1.1)	2.0 (1.3,2.1)	< 0.001
Hemoglobin (HGB) (g/L)	115 (76,133)	124 (112,126)	136 (130,144)	0.003
Platelet count (×10^9/L)	179 (116,247)	214 (202,264)	241 (170,279)	0.187
ALT (U/L)	31 (19,43)	21 (13,30)	20 (15,22)	0.070
TBIL (μmol/L)	9.7 (7.0,16.2)	7.4 (3.9,9.6)	9.8 (6.1,11.4)	0.206
Albumin (g/L)	32.1 (28.8,36.4)	31.1 (28.9,33.0)	42.2 (38.6,46.3)	< 0.001
Creatinine (μmol/L)	57.9 (51.2,66.8)	59.0 (43.5,68.3)	58.0 (49.0,65.7)	0.756
Serum potassium (mmol/L)	4.0 (3.3,4.6)	4.0 (3.7,4.3)	4.0 (3.9,4.1)	0.958
Serum sodium (mmol/L)	135 (130,138)	138 (136,140)	139 (138,141)	0.019
Serum chloride (mmol/L)	102 (99,105)	102 (98,105)	105 (104,106)	0.025
CD4^+^T-cell count (cells/μl)	21 (8,36)	27 (10,61)	702 (515,878)	< 0.001
HIV load (log10 copies/ml)	4.5 (4.1,4.8)	4.9 (4.2,5.3)	–	0.223
*CSF parameters*				
ICP (mmH_2_O)	220 (98,310)	90 (75,135)	110 (95,128)	0.036
WBC count (×10^6/L)	21.5 (3.3,91.8)	1.5 (1.0,2.0)	2.0 (1.0,3.0)	0.002
Lymphocyte count (× 10^6/L)	8.0 (1.8,35.3)	1.0 (0.0,2.0)	1.0 (1.0,2.0)	0.001
Glucose (mmol/L)	2.3 (1.7,3.3)	3.1 (2.8,3.8)	3.0 (2.9,3.3)	0.060
Chlorine (mmol/L)	122 (119,123)	123 (120,128)	125 (124,127)	0.010
Total protein (mg/L)	271.6 (112.0,355.1)	172.3 (144.3,225.6)	145.4 (114.5,182.0)	0.111

*HIV* human immunodeficiency virus, *CM* cryptococcal meningitis, *WBC* white blood cell, *ALT* alanine aminotransferase, *TBIL* total bilirubin, *CSF* cerebrospinal fluid, *ICP* intracranial pressure. Data are presented as median [interquartile range, (IQR)] or cases (percentage)

### CSF cytokine levels profile

**Fig. 2:**
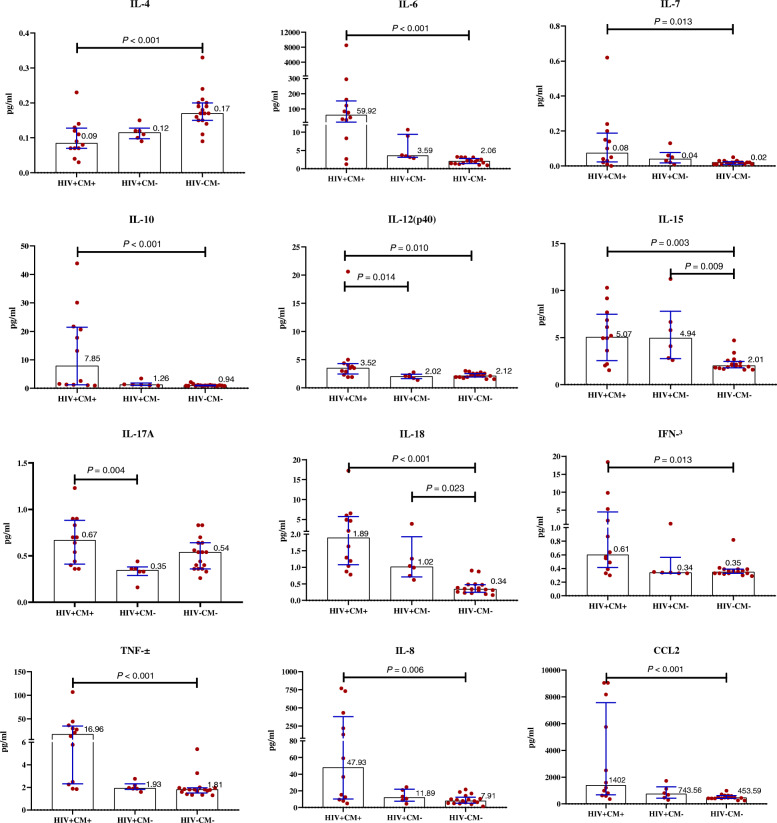
CSF cytokine levels profile among three groups The graph showed IL-4, IL-6, IL-7, IL-10, IL-12(P40), IL-15, IL-17A, IL-18, IFN-γ, TNF-α, IL-8 and CCL2 in CSF at the baseline

At admission, HIV + CM+ group presented higher CSF levels of IFN-γ (*P* = 0.013), IL-6 (*P* < 0.001), IL-7 (*P =* 0.013), IL-8 (*P* = 0.006), IL-10 (*P* < 0.001), IL-12(P40) (*P* = 0.010), IL-15 (*P* = 0.003), IL-18 (*P* < 0.001), CCL2 (*P <* 0.001), and TNF-α (*P <* 0.001) than HIV- CM- group, also had higher CSF levels of IL-12(P40) (*P* = 0.014) and IL-17A (*P* = 0.004) than HIV + CM- group. However, the IL-4 in the CSF of HIV+ CM+ patients was lower than that of HIV-CM- patients (*P <* 0.001) (Fig. 2). There was no significant difference among the three groups of IL-1β, IL-2, IL-5, IL-13, CXCL10, and VEGF-A in CSF.

### Correlation among CSF cytokine concentrations

**Fig. 3:**
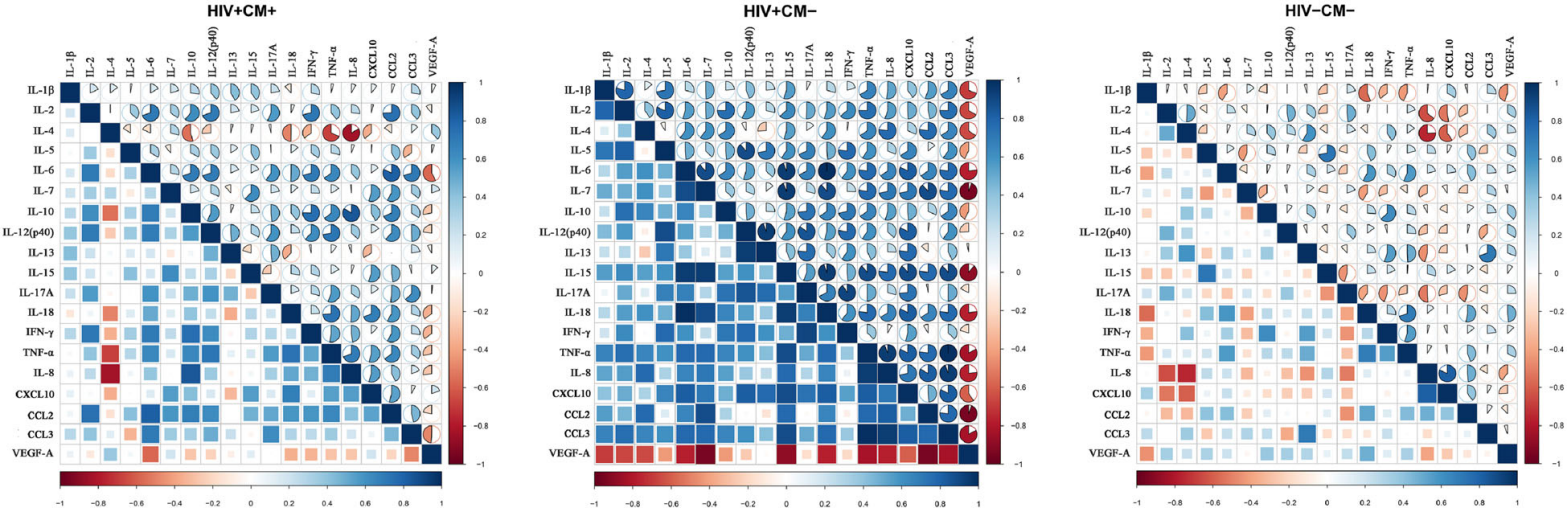
Correlograms of the correlations among 19 CSF cytokine concentrations for the three groups. Blue and red color represent a positive and negative association between the concentrations of the two CSF cytokine that meet at that cell, respectively. The darker and more saturated the color, the greater the magnitude of the correlation. a. In HIV + CM+ group, there were 32 significant correlations among the levels of various cytokines. The median correlation coefficient was 0.263. b. In HIV + CM- group, there were 29 correlations among the levels of various cytokines. The median correlation coefficient was 0.551. c. In HIV-CM- group, there were 22 correlations among the levels of various cytokines. The median correlation coefficient was 0.066

In HIV + CM+ group, there were 32 (18.7%) significant correlations among the levels of various cytokines. In HIV + CM- group and HIV-CM-group, such correlations were 29 (17.0%) and 22 (12.9%), respectively. Although no differences were observed in the numbers of cytokine correlations among the three groups (*P* > 0.05), the CSF cytokine network had undergone major changes. In HIV + CM+ group, when compared with HIV-CM- group, 17 cytokine correlations were lost concomitant with 27 new established correlations; while compared with HIV + CM- group, 12 cytokine correlations were lost concomitant with 27 new established ones. In addition, the intensity of CSF cytokines was also changed among groups. The median correlation coefficients were significantly different between HIV + CM+ and HIV + CM- groups (0.263 vs. 0.551, *P* < 0.001), also between HIV + CM- and HIV-CM- groups (0.551 vs. 0.066, *P* < 0.001) as well (Fig. 3).

### Clinical treatment process and outcome in HIV + CM+ group

Twelve HIV + CM+ patients underwent lumbar puncture and CSF testing at the admission. Except one patient dropped out before treatment, the remaining eleven patients received induction antifungal therapy and CSF testing every 2 weeks. One patient completed the induction therapy and was discharged from the hospital at the week 4. Two patients were passed away as follows: a 54-year-old HIV patient was found to be in the advanced stage of AIDS, with CD4 count of only 8 cells/μl and the CSF pressure measured was over 350mmH_2_O at admission. Although antifungal and lowering intracranial pressure therapy was given, the patient died of intracranial hypertension 3 days after admission. Another 38-year-old patient was initially treated with antifungal therapy effectively, with the CSF fungal culture turned negative after 2 weeks of reexamination. However, the patient continued hematochezia and eventually died of hemorrhagic shock due to the suspected intestinal tuberculosis. Therefore, eight patients survived and were still hospitalized. Thus, the mortality rate was 9.1% (1/11) at week 2 and 18.2% (2/11) at week 4 after initial therapy. The distribution of AIDS patients with CM undergoing cytokine production assays was shown in Fig. 1.

### Changes in CSF cytokine levels during therapy in HIV + CM+ group

CSF cytokine levels were measured at the beginning of the treatment (*n* = 11), week 2 (*n* = 10), and week 4 (*n* = 8) (Table 2). The linear model analysis in the generalized estimating equation shows that compared to the baseline, the levels of IL-2 (*P* = 0.013), IL-13 (*P* = 0.019), IL-17A (*P* = 0.007), VEGF-A (*P* = 0.032) were decreased at week 2, and VEGF-A (*P* = 0.030) at week 4 as well. There were no differences in other cytokines (all *P* > 0.05) in different time point. Also, there was no difference in the levels of all cytokines between survivors and deceased cases (all *P* > 0.05) (Table 3).

**Table 2:** Changes in CSF cytokine levels during therapy

Cytokine (pg/ml)	Baseline (*n* = 11)	Week 2 (*n* = 10)	Week 4 (*n* = 8)
IL-1β	0.10 (0.04,0.98)	0.07 (0.04,0.17)	0.11 (0.04,0.16)
IL-2	0.16 (0.11,0.34)	0.13 (0.12,0.16)	0.14 (0.13,0.23)
IL-4	0.09 (0.07,0.13)	0.09 (0.06,0.14)	0.09 (0.07,0.11)
IL-5	0.90 (0.58,2.73)	1.16 (0.73,1.42)	2.74 (1.00,3.48)
IL-6	43.8 (8.32,162)	7.43 (3.89,17.4)	27.8 (2.98,62.2)
IL-7	0.05 (0.02,0.15)	0.05 (0.03,0.06)	0.07 (0.03,0.09)
IL-10	2.56 (1.22,21.7)	2.19 (1.33,3.87)	2.26 (1.48,4.11)
IL-12(p40)	3.52 (2.89,4.37)	2.89 (1.92,6.58)	3.14 (2.13,5.47)
IL-13	1.50 (1.22,2.89)	1.33 (1.11,1.56)	1.40 (1.24,2.35)
IL-15	4.94 (2.19,7.69)	4.54 (2.81,7.87)	6.30 (2.84,11.2)
IL-17A	0.70 (0.44,0.83)	0.38 (0.33,0.54)	0.48 (0.31,0.77)
IL-18	1.63 (1.04,4.91)	2.40 (1.02,8.97)	2.69 (1.07,11.9)
IFN-γ	0.64 (0.39,5.32)	0.43 (0.32,0.73)	0.46 (0.36,2.59)
TNF-α	20.6 (2.26,36.3)	4.58 (3.11,19.5)	5.81 (4.43,21.2)
IL-8	36.7 (9.44,431)	24.2 (22.8,73.8)	39.7 (21.4,147)
CXCL10	102 (4.16,791)	315 (27.6,780)	261 (24.0,1707)
CCL2	1209 (629,5751)	1079 (851,4060)	2578 (846,5136)
CCL3	0.26 (0.09,12.9)	0.24 (0.08,4.68)	0.32 (0.12,1.08)
VEGF-A	0.52 (0.44,0.55)	0.48 (0.38,0.49)	0.43 (0.29,0.46)

*IL* Interleukin, *IFN* Interferon, *TNF* Tumor Necrosis Factor, *VEGF-A* Vascular Endothelial Growth Factor A. Data are presented as median [interquartile range, (IQR)]

**Table 3:** Linear regression analysis of CSF cytokine in different time and clinical outcome

Cytokine	Item	***β*** (95% ***CI***)	Wald ***χ***^**2**^	***P*** value
**IL-2**	Time			
	Baseline	0^a^		
	Week 2	−0.079(−0.141,-0.017)	6.190	0.013
	Week 4	−0.044(−0.145,0.058)	0.711	0.399
	Outcome			
	Survival	0^a^		
	Death	0.030(−0.044,0.104)	0.622	0.430
**IL-13**	Time			
	Baseline	0^a^		
	Week 2	−1.039(−1.910,-0.168)	5.466	0.019
	Week 4	2.137(−3.364,7.638)	0.580	0.446
	Outcome			
	Survival	0^a^		
	Death	−1.475(−3.066,0.116)	3.304	0.069
**IL-17A**	Time			
	Baseline	0^a^		
	Week 2	−0.209(−0.360,-0.058)	7.327	0.007
	Week 4	−0.145(−0.311,0.021)	2.929	0.087
	Outcome			
	Survival	0^a^		
	Death	0.239(−0.038,0.516)	2.849	0.091
**VEGF-A**	Time			
	Baseline	0^a^		
	Week 2	−0.053(−0.101,-0.005)	4.619	0.032
	Week 4	−0.103(− 0.196,-0.010)	4.713	0.030
	Outcome			
	Survival	0^a^		
	Death	−0.130(−0.293,0.033)	2.456	0.117

*IL* Interleukin, *VEGF-A* Vascular Endothelial Growth Factor A, ^a^ Set to zero because this parameter is redundant

## Discussion

In the current study, we prospectively analyzed the cytokine in CSF of AIDS patients with CM and illustrated a comprehensive image of 19 cytokines after infection. We found in HIV+ CM+ group the higher CSF IFN-γ, TNF-α, IL-6, IL-7, IL-8, IL-10, IL-12 (P40), IL-15, IL-18, CCL2 levels but lower IL-4 when compared with the HIV-CM- group, while a higher level of IL-12 (P40) and IL-17A compared with HIV + CM- group. After receiving the induction antifungal treatment, HIV + CM+ patients’ mortality was 9.1% (1/11) at week 2, and 18.2% (2/11) at week 4. Compared with baseline CSF cytokines, the IL-2, IL-13, IL-17A, VEGF-A, and the VEGF-A levels were decreased in the follow-up. However, there was no difference in all cytokines between survivors and the death in this study.

Generally, the balance of Th1-Th2 cytokines in the host is closely related to microbial infection. Th1 induces the production of IL-1β, IFN-γ, TNF-α to recruit and activate macrophages. It up-regulates reactive oxygen species’ production to kill invading fungi, thereby triggering specific adaptive CD4 + T cell responses. The protective response against cryptococcus is related [22, 23]. On the opposite, Th2 cytokines act as down-regulators of the cellular immune response, inhibit T cell proliferation and are associated with impaired infection control and poor prognosis [15, 24]. Xu et al. found that [25], HIV + CM+ patients had higher levels of CSF cytokines and chemokines than HIV-CM- patients, Th1 cytokines (such as TNF-α, IFN-γ and IL-12), Th2 cytokines (such as IL-4, IL-5, IL-6 and IL-12) and macrophage cytokines (such as IL-8 and CCL2) were particular high indicated the unbalance of Th1-Th2 cytokines in CSF. Most of our results were consistent with the previous study, but we found IL-4 in CSF of HIV + CM+ patients were lower than that of HIV-CM- patients. This lack of IL-4 may occur due to the immune response to cryptococcus of AIDS patients with CM. Research showed that Th2 cells are dominant in the early stages of cryptococcal infection [26]. IL-4 activated macrophages are associated with uncontrolled and severe CM [27]. Moreover, the higher concentrations of IL-4 in CSF before treatment implicate the risk of early death in HIV + CM+ patients [15].

In addition, we found an increased CSF IL-17A in HIV + CM+, which add supporting evidence of the recent studies [28, 29]. IL-17 promotes inflammatory pathology in autoimmune disease but protects the host against many pathogens, particularly antifungal protection [30, 31]. An experimental model of macrophage activation syndrome provides evidence that Th17 cells can be drivers of a cytokine storm that is independent of IFN-γ [32]. In HIV + CM+ who have not yet started ART, increased concentrations of CSF IL-17A combination with other cytokines (IL-10 and classical Th1 cytokines) was associated with an IFN-γ/TNF-α producing cryptococcal antigen-specific peripheral blood T cells response that, in turn, associated with lower 2-week mortality [33].

Sometimes the borderline between the normal response of cytokines to severe infection and the unregulated response is blurred, especially considering that certain cytokines may help control infection and be harmful to the host [34]. The interdependence of these inflammatory mediators further complicates the distinction between normal and dysregulated responses. Mora et al. [35] reported the relationship between cytokines and clinical outcomes in HIV + CM+. At the 2nd and 10th week of survivors, the CSF levels of IL-8, IL-12p40, IL-17A, TNF-α, INF-γ and serum TNF-α were significantly increased. Patients with increased ICP at the 10th week showed a high level of CSF IL-10 associated with a poor prognosis. Subsequently, the team reported again on the relationship between the dynamic changes of serum and CSF cytokines in HIV + CM+ and the clinical outcome [36]. They observed gradual changes in the expression of cytokines that contribute to the Th1 pattern. However, Jarvis et al. put forward a different point of view in their study [15]. They stated that in the CSF of HIV + CM+ patients, several other cytokines such as IL-2, IL-4, IL-6, IL-8, IL-10, IL-17, IFN-γ, TNF-α and CCL2 participate in the immune response, among which Th1 (IFN-γ), Th2 (IL-4 and IL-10) and Th17 (IL-17) had a synergistic effect. And these changes were positively correlated with the rapid clearance of cryptococcus in CSF and the clinical outcome during treatment. In this study, we also found the concentrations of Th1, Th2 and Th17 cytokines were closely related to each other. The existence of this comprehensive inflammatory response was conducive to clinical outcomes, rather than harmful [15]. Due to these different viewpoints, there are few recommendations for immunoadjuvant therapy of AIDS patient with CM [37]. The immune pathogenesis of HIV + CM+ still needs further exploration.

In fact, the balance of pro-inflammatory and anti-inflammatory stimuli is essential for effective control of fungal infections [38, 39]. The ideal immune response to cryptococcal infection relies on a strict balance between Th1, Th2 and Th17 to restrict fungal growth while preventing excessive tissue damage and immunopathology [40]. Research showed that bolstering immunity to reduce pathogen burden may be unsuccessful in cases of defective tolerance with significant tissue and/or organ damage, the collateral damage caused by the immune response as it attempts to clear the pathogen can be more deadly than the pathogen itself, while immunomodulation may be beneficial [41, 42]. The immunomodulatory mechanism that controls the intensity and duration of the host response is one of the main strategies that may provide infection tolerance and maintain host fitness and homeostasis [43, 44].

The formation of coordinated complex networks of cytokines has been widely accepted. HIV and intracranial cryptococcal infection have caused major changes in the interconnection between functionally different classes of cytokines. Consistent with previous findings [45, 46], the correlation between most cytokines in the HIV + CM- group is significantly enhanced, reflecting the regulatory effect of HIV infection on cytokines. However, this correlation among cytokines weakens when CM occurs, which implicates an independent effect of CM on CSF cytokines. Although neither various functions of cytokines nor the correlations of their direct impact on the immune response were clear, our study showed that no matter in the process of HIV infection or CM infection, cytokine was up or down-regulated in CSF. Our research constructed a comprehensive image of 19 cytokines after infection and provided direct evidence for the relationship between the pathogenicity of *Cryptococcus neoformans* and cytokines. It also offers new ideas for the treatment of AIDS combined with CM. Under certain conditions, it can co-stimulate or antagonize the production and secretion of specific cytokines, which resisting cryptococcosis or protecting the body.

Although we present some pertinent findings, the limitations of our study must also be acknowledged. The number of patients afflicted with CM is relatively small that lead to a small sample size. And the follow-up period is limited to the length of the hospitalization. We will further carry out the follow-up in multi centers.

## Conclusion

In this study, the HIV + CM+ patients showed higher CSF levels, including IFN-γ, IL-6, IL-7, IL-8, IL-10, IL-12 (P40), IL-15, IL- 18, CCL2 and TNF-α than HIV-CM- participants, and higher IL-12 (P40) and IL-17A levels than HIV + CM-subjects. After the antifungal therapy, the mortality of HIV + CM+ patients were 9.1% (1/11) at week 2. The mortality raised to 18.2% (2/11) at week 4, with the IL-2, IL-13, IL-17A, VEGF-A and the VEGF-A in CSF decreased. However, there was no difference in the levels of all cytokines between survivors and the dead. Our study described the CSF cytokine network in AIDS patients with CM, providing new clues and strategies for these patients.

## Supplementary Information

**Additional file 1: Fig. S1** Cytokines in CSF and plasma of two patients.

## Data Availability

The datasets used and/or analyzed during the current study are available from the corresponding authors (lilydaier@ccmu.edu.cn; whdoc@ccmu.edu.cn) on reasonable request.

## Abbreviations

AIDS: Acquired immunodeficiency syndrome
ART: Antiretroviral therapy
ALT: Alanine aminotransferase
CM: Cryptococcal meningitis
CSF: Cerebrospinal fluid
HIV: Human immunodeficiency virus
HIV + CM +: AIDS patients with CM
HIV + CM-: AIDS patients without CM
HIV-CM-: Non-AIDS and non-CM patients
IQR: Interquartile range
ICP: Intracranial pressure
IRIS: Immune reconstitution inflammatory syndrome
SPSS: Statistical Package for the Social Sciences
TBIL: Total bilirubin
WBC: White blood cell
