# Supplementary figures and images for: A preliminary study on the characteristics of Th1/Th2 immune response in cerebrospinal fluid of AIDS patients with cryptococcal meningitis
Source: BMC Infect Dis. 2021 May 29;21:500. doi: 10.1186/s12879-021-06138-z (PMC8164222; doi:10.1186/s12879-021-06138-z)

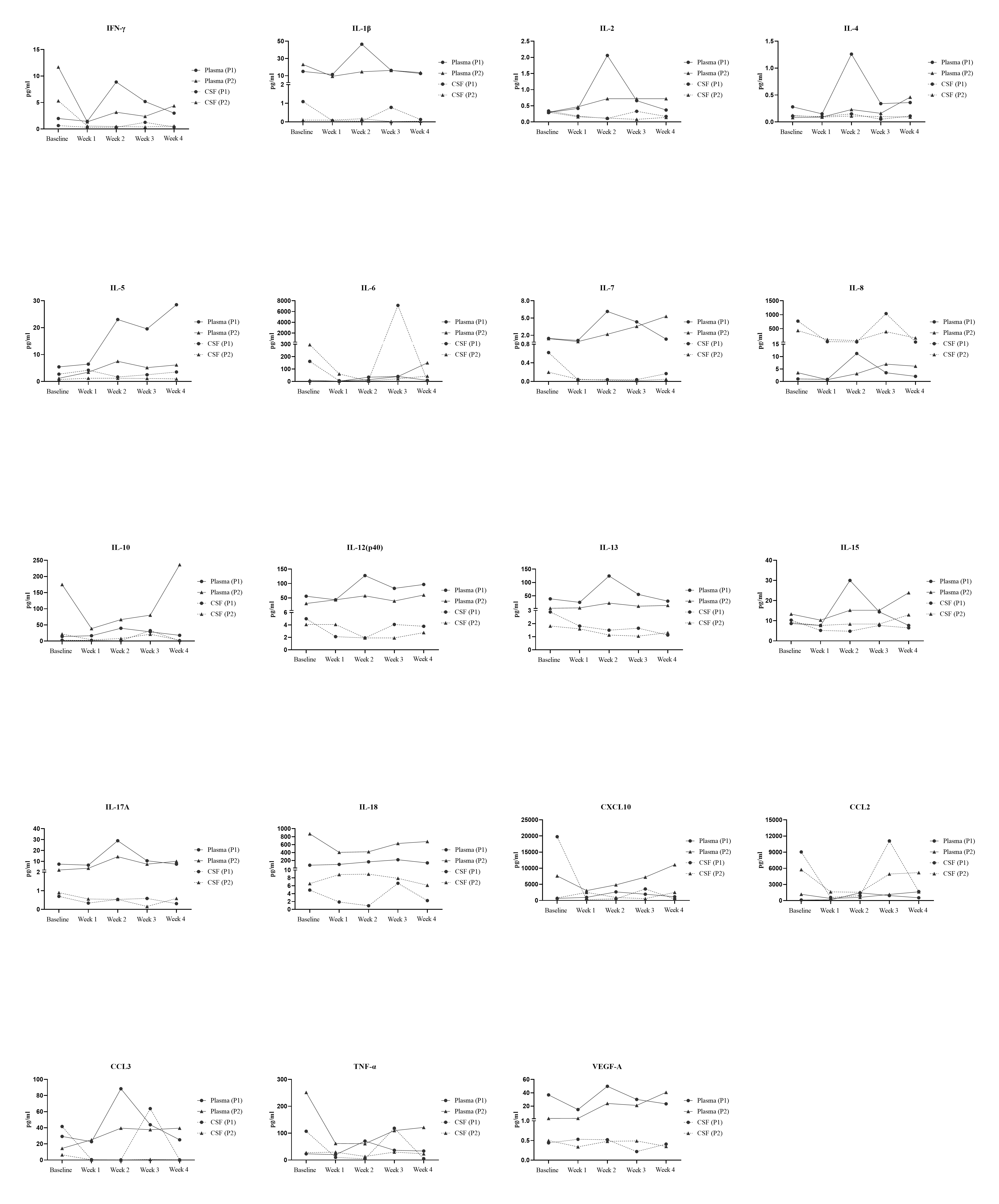

Supplement: Supplementary file 1 — Additional file 1: Fig. S1 Cytokines in CSF and plasma of two patients. [file 12879_2021_6138_MOESM1_ESM.tif]
